# The NADPH oxidase NOX2 mediates loss of parvalbumin interneurons in traumatic brain injury: human autoptic immunohistochemical evidence

**DOI:** 10.1038/s41598-017-09202-4

**Published:** 2017-08-18

**Authors:** Stefania Schiavone, Margherita Neri, Luigia Trabace, Emanuela Turillazzi

**Affiliations:** Department of Clinical and Experimental Medicine, University of Foggia, Via Napoli 20, 71122 Foggia, Italy

## Abstract

Pharmacological interventions for traumatic brain injury (TBI) are limited. Together with parvalbumin (PV) loss, increased production of reactive oxygen species (ROS) by the NADPH oxidase NOX enzymes represents a key step in TBI. Here, we investigated the contribution of NOX2-derived oxidative stress to the loss of PV immunoreactivity associated to TBI, performing immunohistochemistry for NOX2, 8-hydroxy-2′-deoxyguanosine (8OHdG) and PV on *post mortem* brain samples of subjects died following TBI, subjects died from spontaneous intracerebral hemorrhage (SICH) and controls (CTRL). We detected an increased NOX2 expression and 8OHdG immunoreactivity in subjects died from TBI with respect to CTRL and SICH. NOX2 increase was mainly observed in GABAergic PV-positive interneurons, with a minor presence in microglia. No significant differences in other NADPH oxidase isoforms (NOX1 and NOX4) were detected among experimental groups. NOX2-derived oxidative stress elevation appeared a specific TBI-induced phenomenon, as no alterations in the nitrosative pathway were detected. Our results suggest that NOX2-derived oxidative stress might play a crucial role in the TBI-induced loss of PV-positive interneurons.

## Introduction

Traumatic brain injury (TBI) represents a dramatic health problem, being one of the leading cause of disability^1^ and mortality^2^. TBI is commonly and most basically defined as an alteration in brain functioning or the emergence of brain pathology caused by an external force^3^. In head injury, different pathophysiological mechanisms occur which are set in motion by the injury event and take time to evolve^4^. In fact, the pathophysiology of TBI is extremely complex and heterogenous. Traditionally, “primary” versus “secondary” damage has been distinguished. However, over the years, it has become increasingly clear that brain injury may be considered a process beginning with an impact rather than a single event that may be then followed by secondary complications^4^. In this ongoing process, overlapping and interrelated phenomena are intertwined^5^ and, following the primary mechanical damage, different neurodetrimental processes (secondary, non-mechanical damage) representing the pathophysiological consequences started at the time of the initial injury, occur^6^. These can result in several neurodetrimental effects, such as reduced blood flow and oxygen metabolism, metabolic deregulation and reduction in oxidative metabolism, glutamate-induced excitotoxicity, mitochondrial dysfunction and accumulation of reactive oxygen species (ROS) with a global increase of oxidative stress^7–11^. This phenomenon, resulting from a disequilibrium between the functioning of ROS-generating systems and antioxidants, has been widely reported as one of the key contributors to the development of TBI damage^12^. Although mitochondria have been described as the main source of ROS^13^, several evidences have pointed out a crucial role of the NADPH oxidase NOX enzymes to generation of superoxide, the most commonly occurring cellular free radical^14^. This enzymatic family, composed of several NOX isoforms distributed in a large variety of cells and tissues, has been shown to crucially contribute to different physiological and pathological functions^14^. In particular, NOX2 enzyme is known to regulate specific physiological pathways in the central nervous system (CNS)^15^ and to be a key player in the pathogenesis of CNS disorders, going from neurodegenerative^16, 17^ to psychiatric diseases^18, 19^. Recent reports described a key role of NADPH oxidase elevations in experimental rodent models of TBI, such as the “moderately severe weight-drop impact head injury” mouse model^20^, also supported by the beneficial effects of pharmacological or genetic NOX enzyme inhibition on TBI-induced neuronal damage^21, 22^. In particular, it has been demonstrated that intraperitoneal apocynin administration to rats before TBI was able to decrease ROS production, as well as prevent blood brain barrier disruption and microglia activation^22^. Moreover, apocynin treatment, immediately after TBI, determined a reduction of inflammatory and oxidative damage, without any protective effect on the development of brain edema^23^. TBI-induced increase in the levels of malondialdehyde was also prevented by apocynin pretreatment^20^. Dysfunctions of GABAergic neurotransmission, another important pathological pathway leading to neuronal impairment following TBI, mainly occur through damage to parvalbumin (PV)-immunoreactive interneurons, in terms of loss and altered activity of this neuronal subtype^24^. However, the leading cause of decreased PV-positive neurons in TBI has not been clarified yet. Here, we investigated the possible contribution of the NADPH oxidase NOX2-derived oxidative stress to the loss of PV immunoreactivity in human subjects died following TBI compared to subjects died from spontaneous intracerebral hemorrhage (SICH) and controls (CTRL). *Post mortem* brain samples of these subjects were analyzed for expression of NOX2, 8OHdG, PV and markers of nitrosative stress by immunohistochemistry. NOX2 expression in cellular brain subpopulations, i.e. neurons, in particular PV-immunoreactive interneurons, microglia and astrocytes was also evaluated.

## Results

### Increase of NOX2 and 8OHdG immunostaining in the cortex of subjects died following TBI

**Figure 1:**
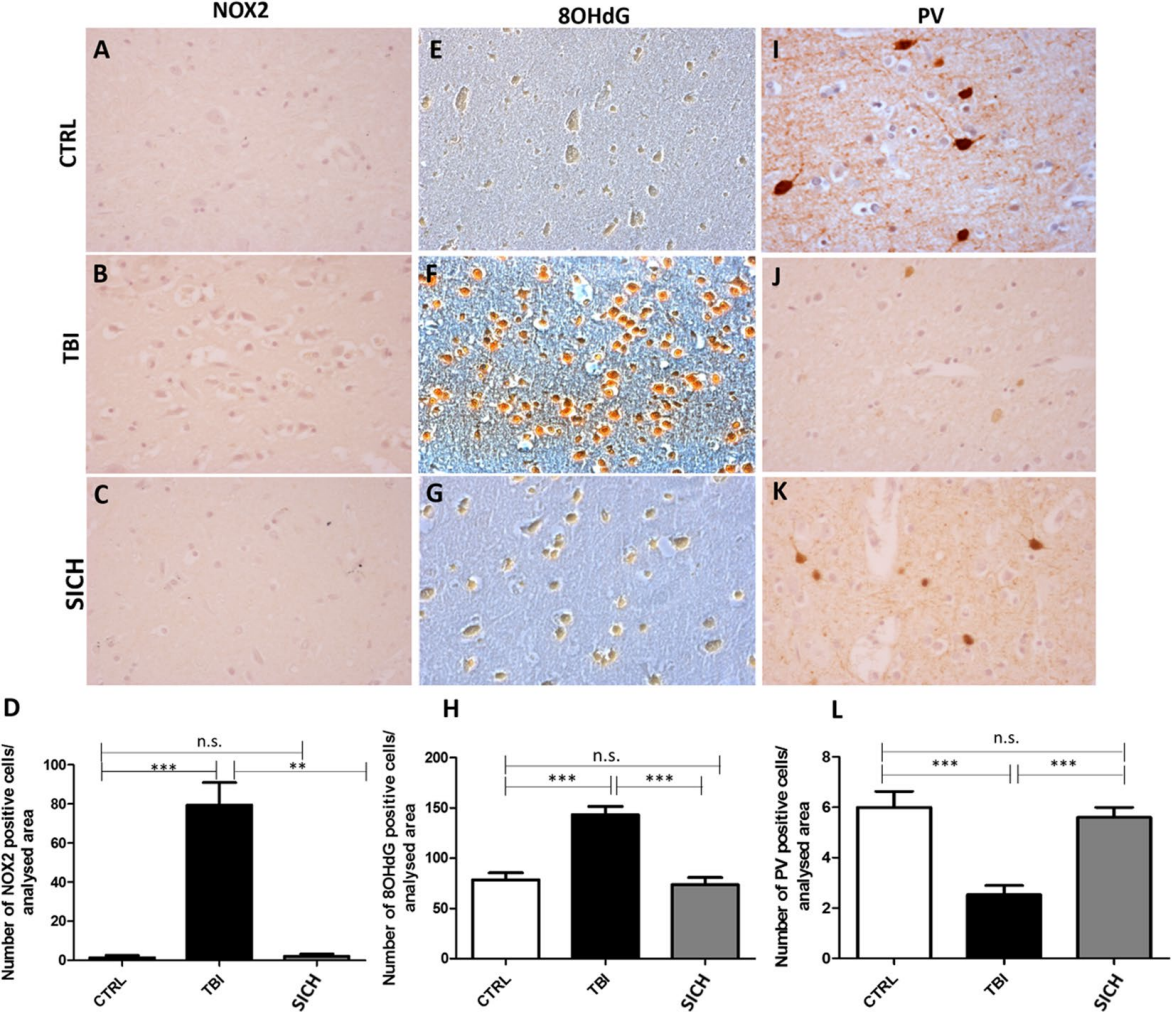
Increase of oxidative stress and loss of PV-positive interneurons in the cortex of subjects died following traumatic brain injury. (**A**–**C**) Representative images of NOX2 immunostaining in the cortex of controls (CTRL, n = 5) (**A**), subjects died following TBI (TBI, n = 15) (**B**) and subjects died following spontaneous intracerebral hemorrhage (SICH, n = 5) (**C**). (**D**) Quantification of NOX2-positive-stained cells in the cortex of controls (CTRL, n = 5), subjects died following TBI (TBI, n = 15) and subjects died following spontaneous intracerebral hemorrhage (SICH, n = 5). Results are expressed as means ± s.e.m. One-way ANOVA, followed by Tukey’s post hoc test, F = 14.36; **P < 0.01; ***P < 0.001; n.s. = not significant. (**E**–**G**) Representative images of 8OHdG immunostaining in the cortex of controls (CTRL, n = 5) (**E**), subjects died following TBI (TBI, n = 15) (**F**) and subjects died following spontaneous intracerebral hemorrhage (SICH, n = 5) (**G**). (**H**) Quantification of 8OHdG-positive-stained cells in the cortex of controls (CTRL, n = 5), subjects died following TBI (TBI, n = 15) and subjects died following spontaneous intracerebral hemorrhage (SICH, n = 5). Results are expressed as means ± s.e.m. One-way ANOVA, followed by Tukey’s post hoc test, F = 17,72; ***P < 0.001; n.s. = not significant. (**I**–**K**) Representative images of PV immunostaining in the cortex of controls (CTRL, n = 5) (**I**), subjects died following TBI (TBI, n = 15) (**J**) and subjects died following spontaneous intracerebral hemorrhage (SICH, n = 5) (**K**). (**L**) Quantification of PV-positive-stained cells in the cortex of controls (CTRL, n = 5), subjects died following TBI (TBI, n = 15) and subjects died following spontaneous intracerebral hemorrhage (SICH, n = 5). Results are expressed as means ± s.e.m. One-way ANOVA, followed by Tukey’s post hoc test, F = 18,20; ***P < 0.001; n.s. = not significant.

In order to investigate whether NOX2-derived oxidative stress might be involved in TBI-induced neuropathological alterations, we performed immunohistochemical analysis for NOX2 expression in the cortex of subjects died following TBI, compared to subjects died from SICH and CTRL. While NOX2 immunoreactivity was detected in very few cells of the frontal cortex of SICH subjects and CTRL, a significant elevation of the number of NOX2 positive cells was observed in subjects died following TBI (Fig. 1A–D, One-way ANOVA, followed by Tukey’s post hoc test, F = 14.36; **P < 0.01; ***P < 0.001; n.s. = not significant). The same results were found for 8OHdG immunoreactivity evaluation (Fig. 1E–H, One-way ANOVA, followed by Tukey’s post hoc test, F = 17,72; ***P < 0.001; n.s. = not significant). Moreover, in our experimental conditions, the observed increased NOX2 expression in the frontal cortex of TBI subjects appears to be specific with respect to other NADPH oxidase isoforms, such as NOX1 and NOX4. Indeed, immunohistochemical analysis and pertaining quantifications showed the presence of a weak basal NOX1 staining in CTRL which did not significantly differ from the staining detected in TBI and SICH subjects (Suppl. Fig. 2A–C and G; One-way ANOVA, followed by Tukey’s post hoc test, F = 0,008367; P = 0,8332). The same was observed for NOX4 expression (Suppl. Fig. 2D–F and H One-way ANOVA, followed by Tukey’s post hoc test, F = 0,4614; P = 0,6364).

**Figure 2:**
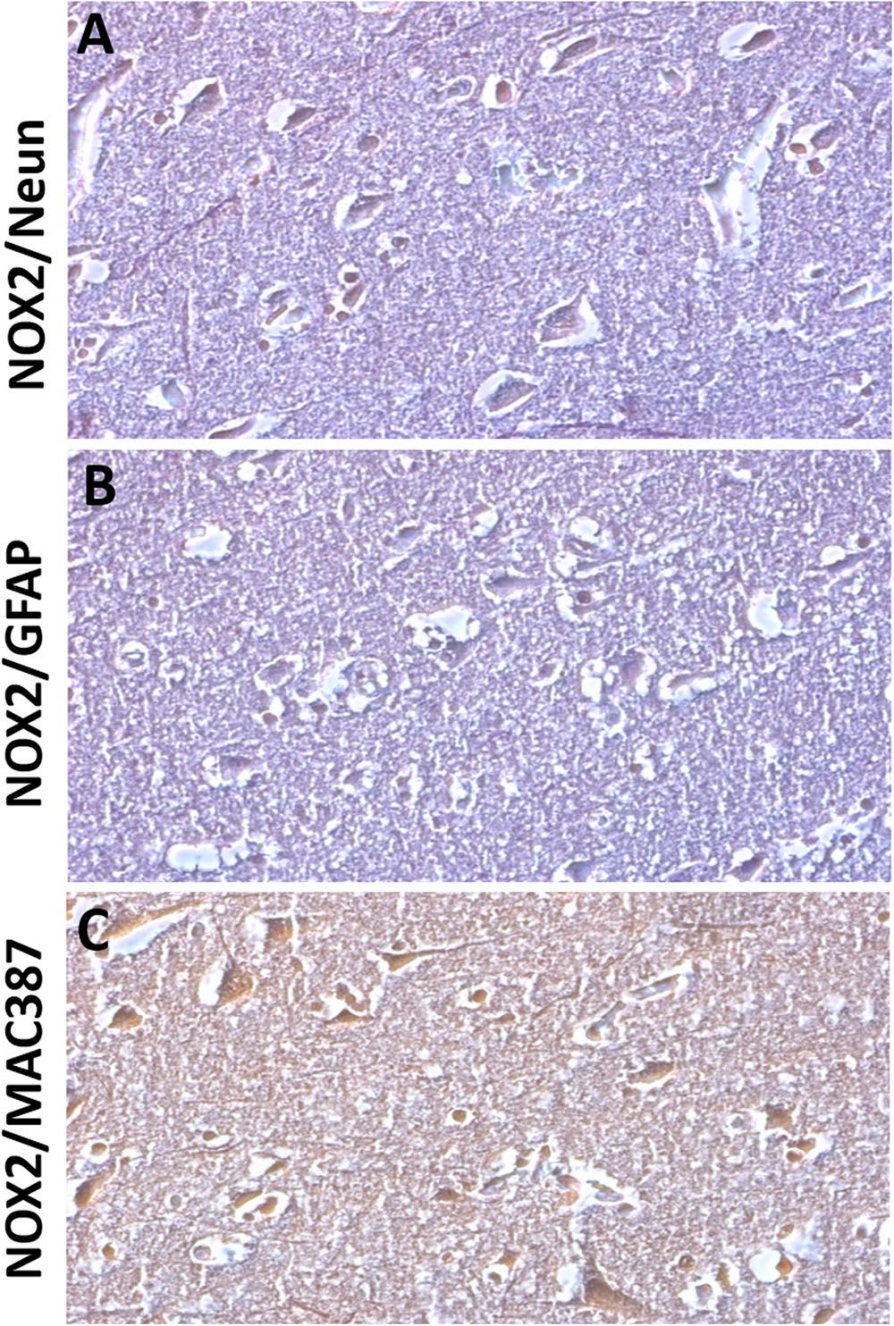
NOX2 increase in cortical neurons and microglia of subjects died following TBI. (**A**–**C**) Representative contrast phase images of double immunostaining for NOX2/Neun (**A**), NOX2/GFAP (**B**) and NOX2/MAC387 (**C**) in the cortex of subjects died following TBI (n = 15).

### TBI-induced oxidative stress increase was associated to cortical PV-positive interneurons decrease

In order to evaluate if TBI-induced increase in NOX2 expression might be associated to decreased number of cortical PV-positive interneurons, we performed immunohistochemical analysis for PV-immunoreactivity in the cortex of subjects died following TBI, compared to subjects died following SICH and CTRL. No differences were detected in the number of PV-positive cells between subjects died following SICH and CTRL, while a marked decrease of this protein immunoreactivity was observed in subjects died from TBI (Fig. 1I–L, One-way ANOVA, followed by Tukey’s post hoc test, F = 18,20; ***P < 0.001; n.s. = not significant).

### NOX2 immunostaining was increased in the cortical GABAergic PV-positive interneurons of subjects died following TBI

**Figure 3:**
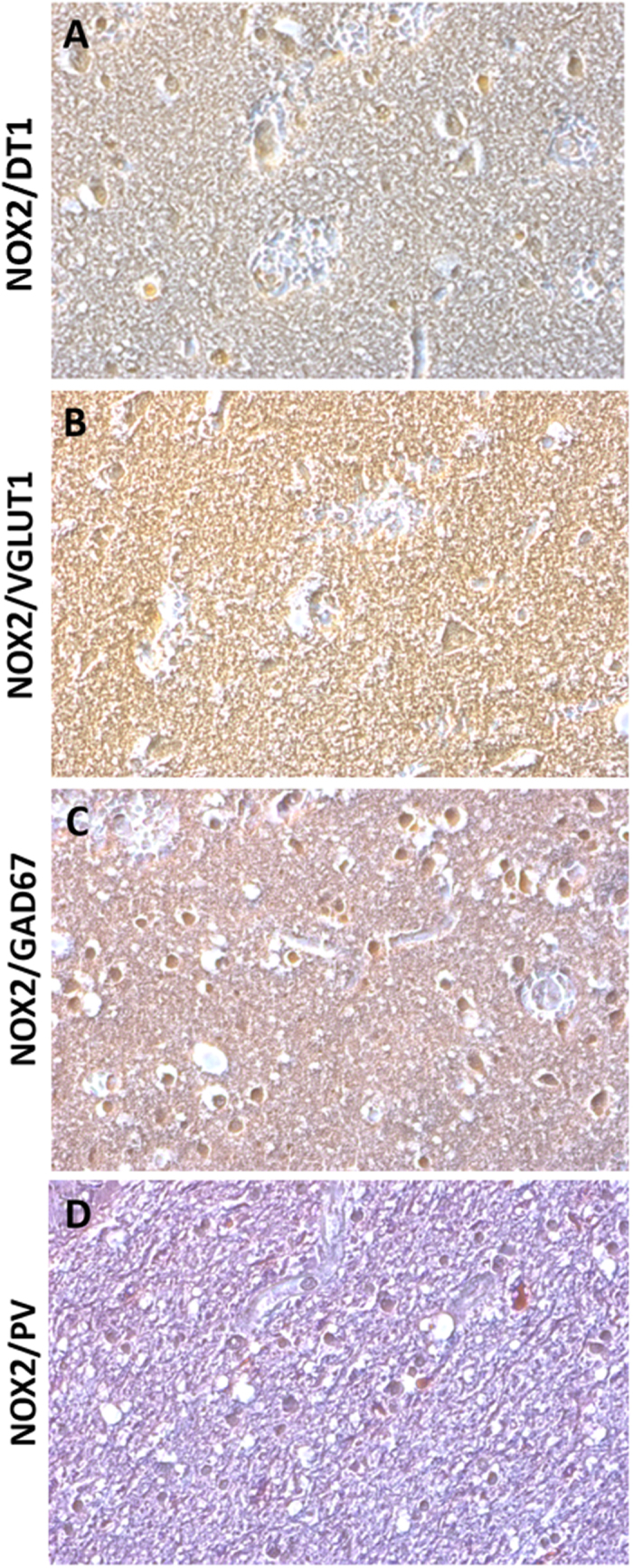
NOX2 increase in cortical GABAergic PV-positive interneurons. (**A**–**D**) Representative contrast phase images of double immunostaining for NOX2/DT1 (**A**), NOX2/VGLUT1 (**B**), NOX2/GAD67 (**C**) and NOX2/PV (**D**) in the cortex of subjects died following TBI (n = 15).

To investigate which brain cellular subpopulation was involved in NOX2 elevation, we performed double immunohistochemistry for NOX2, Neun, and GFAP. NOX2 and MAC387 were also investigated. NOX2 immunoreactivity was mainly found in Neun-positive cells (Fig. 2A), whereas virtually no NOX2 co-staining was detected in GFAP positive cells (Fig. 2B). The presence of co-stained NOX2/MAC387 cells indicated that microglia was also involved in the observed NOX2 elevation (Fig. 2C). To identify the neuronal subtype specifically implicated in NOX2 increase, double immunohistochemistry for NOX2 and DT1, NOX2 and VGLUT1, NOX2 and GAD67, as well as NOX2 and PV was performed. While a weak NOX2 co-staining was detected in DT1 and VGLUT1 immunoreactive cells (Fig. 3A,B), a marked co-expression of NOX2 and GAD67 was observed in the cortex of subjects died following TBI (Fig. 3C). Importantly, NOX2 immunoreactivity was detected in the PV-positive subtype of GABAergic neurons (Fig. 3D).

### Nitrosative stress was not affected by TBI

**Figure 4:**
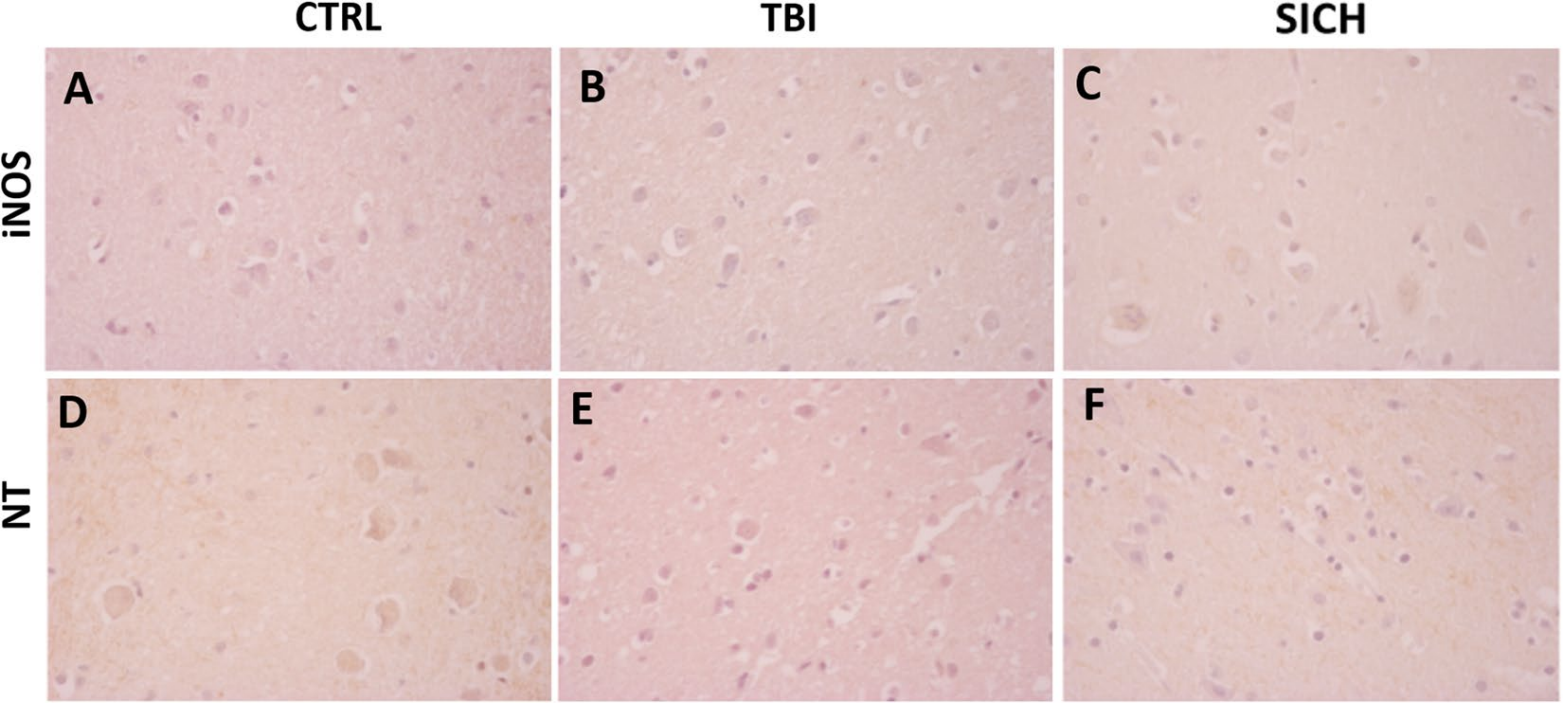
The nitrergic system is not affected by TBI. (**A**–**C**) Representative images of iNOS immunostaining in the cortex of controls (CTRL, n = 5) (**A**), subjects died following TBI (TBI, n = 15) (**B**) and subjects died following spontaneous intracerebral hemorrhage (SICH, n = 5) (**C**). (**D**–**F**) Representative images of NT immunostaining in the cortex of controls (CTRL, n = 5) (**D**), subjects died following TBI (TBI, n = 15) (**E**) and subjects died following spontaneous intracerebral hemorrhage (SICH, n = 5) (**F**).

In order to investigate if TBI specifically increased NOX2-derived oxidative stress or determined a non-specific elevation of oxidative and nitrosative pathways, we performed immunohistochemical analysis for iNOS and nitrotyrosine (NT), in the cortex of subjects died following TBI, SICH and CTRL. Virtually no iNOS-positive cells were counted in the three groups (Fig. 4A–C) and the same was observed for NT immunoreactivity (Fig. 4D–F).

## Discussion

In this study, we investigated the possible contribution of NOX2-derived oxidative stress to neuropathological alterations associated to TBI, with a particular focus on the loss of PV-positive interneurons. We found that expression of NOX2 was significantly increased in the cortex of subjects died following TBI, with respect to subjects died from SICH and CTRL. The same was observed for the expression of 8OHdG, one of the predominant form of ROS-induced oxidative damage to DNA^25^, which has therefore been widely used as a very reliable biomarker for oxidative stress presence and previously found increased in preclinical and clinical investigations on central and peripheral neurological disorders as well as psychiatric illnesses^26–31^. Our results are in line with recent findings reporting increased oxidative stress after TBI^32, 33^. Indeed, in a recent work, Lorente and co-workers demonstrated that total antioxidant capacity and lipid peroxidation state were strictly related to the mortality rate in TBI^34^. Consistent with these findings, other markers of oxidative stress such as thiobarbituric acid-reactive substances, 4-hydroxy-2-nonenal, 4-hydroxy-2-hexenal and isoprostanes have been shown as significantly increased in human plasmatic samples derived from TBI subjects^35, 36^. Protein carbonylation, another important marker of oxidative stress, has been also reported in a mouse model of TBI^37^. In this context, Harmon and co-workers recently demonstrated that TBI leads to mitochondrial disruption, in terms of induction of specific microRNA (miR-21 and miR-155), with consequent increased ROS production in the striatum of a rodent model of head injury^38^. Furthermore, current literature regarding the use of antioxidant therapies in treating TBI, including dexanabinol, amino acids, vitamins C and E, progesterone, N-acetylcysteine and enzogenol highlights the efficacy of these treatments in attenuating the oxidative stress induced by TBI^39, 40^. Interestingly, a report by Yilmaz and collaborators describes the beneficial effects of mannitol and hypertonic saline therapy in reducing TBI cellular damage by increasing levels of antioxidant enzymes such as catalase and glutathione peroxidase^41^.

**Figure 5:**
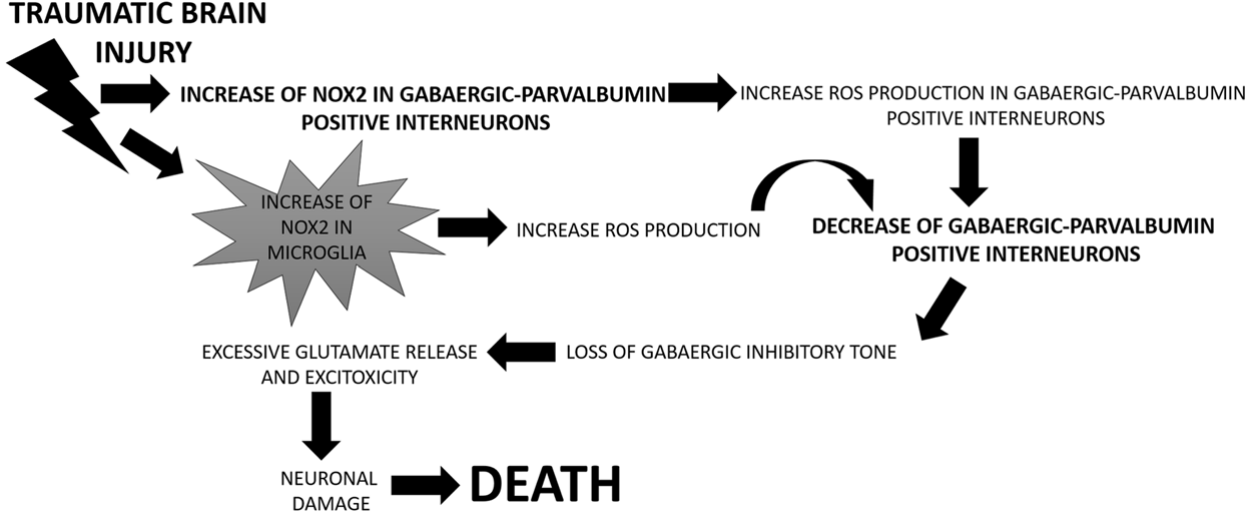
Hypothetical molecular mechanism linking NOX2-derived oxidative stress increase and TBI-induced dysfunctions of GABAergic and glutamatergic neurotransmission. TBI might cause an increase of NOX2 expression in GABAergic PV-positive interneurons with consequent oxidative damage-induced neuronal death. The decrease in GABAergic PV-determines the loss of the inhibitory tone, leading to excitoxicity. In addition, increased NOX2 expression in microglial cells might enhance ROS amount, further damaging PV-positive interneurons.

To the best of our knowledge, this is the first report evaluating NOX2 expression in human subjects died following severe TBI. Indeed, although several studies on rodents described an involvement of the NADPH oxidase in TBI development^42^, as well as the beneficial effects of treatment with apocynin, an antioxidant/NOX inhibitor compound, or of NOX2 deficiency on TBI-induced neuronal damage^20, 43^, only one report described NOX2 and NOX4 increase in neurosurgical brain tissue samples derived from alive human subjects, correlating negatively both NOX2 and NOX4 positive immunoreactivity with the Glasgow Coma Scale of these subjects^44^. We also demonstrated that, together with increased NOX2 expression and oxidative stress, cortical PV-immunoreactivity was reduced in subjects died following TBI. Recent findings highlight the crucial role of dysfunctions of PV-positive interneurons, in terms of both loss and impaired activity, in the development of brain damage induced by traumatic injury^45, 46^. However, these studies have been mainly realized on rodent models of TBI. The observed decrease in PV-immunoreactivity is in line with a paper of Buriticá and co-workers performed on human cortical contusion tissue, describing changes in PV-positive interneurons amount in layer II^47^. In the present study, we showed that NOX2 elevation mainly occurs in neurons with a minor presence of NOX2 immunoreactivity in microglial cells. Our results are in line with previous observations obtained on mice, reporting that, following TBI, the NADPH oxidase expression and activity exhibit a biphasic elevation in the cerebral cortex and hippocampus, with an early peak of increase in neurons, followed by a second phase in which NOX2 expression is also detectable in microglial cells^21^. In the same line, Cooney *et al*. reported that increased NOX2 expression occurs in neurons and microglia and that inhibition of NOX, and more specifically NOX2, might decrease pro-inflammatory activity in microglia^48^. ROS production by NADPH oxidase, as well as the number of degenerating neurons in the hippocampal CA3 region and microglial activation after TBI, were also inhibited by apocynin administration^22^. In support of this, Kumar and collaborators recently showed, in an experimental model of TBI, that NOX2 is highly up-regulated in infiltrating macrophages after injury and that NOX2 deficiency reduces the expression of markers of microglia activation, limiting brain tissue degeneration and improving motor recovery^49^. Importantly, here, we showed an increased NOX2 immunoreactivity in GABAergic neurons and, in particular, in PV-positive interneurons. This is a crucial finding of this study, allowing us to hypothesize a possible molecular mechanism linking neuronal injury, NOX2-derived oxidative stress increase and TBI-induced dysfunctions of GABAergic and glutamatergic neurotransmission. Indeed, TBI might induce an increase of NOX2 expression in GABAergic PV-positive interneurons with consequent ROS production elevation in this cortical cellular subtype and oxidative damage, causing neuronal death. The decrease in GABAergic PV-positive interneurons is responsible for the loss of the inhibitory tone, leading to excessive glutamate release and consequent excitoxicity-induced neuronal death. Increased NOX2 expression in microglial cells might enhance ROS amount that can further damage the PV-positive interneurons, contributing to their degeneration and loss (Fig. 5). This hypothesized molecular mechanism is supported by previous preclinical findings on the ketamine model of psychosis reporting a similar mechanism linking the loss of phenotype of fast-spiking PV-positive interneurons, NADPH oxidase increase and dysfunctions of GABAergic and glutamatergic neurotransmission^50, 51^. Moreover, recent reports on human *post mortem* brain samples of suicidal subjects as well as of a cocaine abuser died following excited delirium syndrome identify GABAergic neurons as the most implicated in the increase of NOX2-derived ROS production^26, 27^. Interestingly, at least in our samples, we detected no differences in iNOS and NT immunoreactivity among the different groups, therefore suggesting a specific effect of TBI on oxidative but not nitrosative stress. Contrasting results regarding the effects of TBI-induced nitric oxide (NO) production have been reported^52^. Indeed, some studies on rodents showed an enhancement of the nitrergic system after TBI and a decrease of neuronal necrosis after aminoguanidine administration^53, 54^. In contrast, recent consistent findings point towards a protective role of the nitrergic pathways against TBI-induced neuronal damage. In this line, prolonged aminoguanidine treatment have been demonstrated to exacerbate brain injury in rats. Furthermore, brain injured iNOS knock-out mice showed worse functional outcome than wild type mice^55^. Rangel-Castilla and co-workers described a two-step model of NO metabolism after TBI, including a phase of immediate increase in NO levels after TBI, followed by a later period of decrease^56^. Beyond a possible neuroprotective effect, the absence of concomitant nitrergic pathway alterations observed in our study is also supported by a very recent study reporting a pathogenetic association between TBI-induced increase of NO levels and impairment of mitochondrial respiratory chain^57^. Another crucial finding of the present work is the absence of NOX2-derived oxidative stress increase in subjects died following SICH, indicating that TBI effects on NADPH oxidase are not non-specific findings. This result might be considered in apparent contradiction with previous findings on rodents reporting an association between intracerebral hemorrhage-induced brain injury and enhanced expression of the gp91phox subunit of the NADPH oxidase^58^. However, beyond the impossibility of a direct translation of a study on rodents to human investigations, this previous work was performed on mice at 20-35 weeks of age, therefore mimicking an older patient population, while the subjects died following SICH included in our study did not cover this age range. Moreover, in support of our observations, neuronal injury induced by SICH has been related to mitochondrial dysfunctions^59, 60^. A limitation of the present study is represented by the lack of a direct NOX activity measurement in these human brain samples. With respect to this missing aspect, although it represents certainly a crucial step in the understanding of physiological and pathological roles of the NADPH oxidase, no efficient and reliable methods to directly measure NOX activity in the brain are actually available^61^.

In conclusion, our study performed on human *post mortem* brain samples suggests a crucial and specific role of NOX2 enzyme in the development of early neuropathological alterations induced by TBI, and may represent a significant progress in the understanding of the multiple cellular and subcellular mechanisms occurring in severe TBI^62^. An important goal common both to clinicians and pathologists is to highlight the cascade of molecular pathways involved in the onset and progression of TBI injury with the aim of developing more targeted and specific pharmacologic interventions to improve the outcomes of TBI. Hence, although many existing preclinical studies have tested the therapeutic efficacy of several compounds in animal models by targeting specific pathways leading to neuronal injury (including calcium channel blockers, corticosteroids, excitatory amino acid inhibitors, NMDA receptor antagonists and free radical scavengers^63^), all these pharmacological approaches did not hit the target^64^ and did not show satisfactory clinical success so far, probably because of their focusing on single events, rather than taking the heterogeneous TBI pathology into account^65^. In this context, the identification of the NADPH oxidase NOX2 as crucial and specific molecular agent leading to TBI-associated neuropathological alterations, such as the loss of PV interneurons, might open the way to several significant clinical implications such as a possible and attractive therapeutic use of selective NOX2 inhibitor compounds in TBI and/or the early identification of patients with a worst follow-up or prognosis. Indeed, the significant contribution of NOX2 to TBI-associated neuropathology might be considered as a reliable biomarker to be also used in clinics for the identification of individual differences in the pharmacological response. Further research in this area is clearly needed, especially regarding the possibility to directly measure ROS production (superoxide and/or H_2_O_2_) by NOX2 enzyme in biological samples and unfixed brain tissue of TBI subjects or to investigate possible treatment-related NOX2 expression changes. A time-course analysis of NOX2 expression in subgroups of patients, at different time points from TBI, would be also an important future direction for works in this area.

## Methods

### Case selection

Cases were selected from the case series of the section of Legal Medicine, Department of Clinical and Experimental Medicine, University of Foggia, Italy. A total of 15 patients with severe TBI were studied (men and women). The mean age was 38 ± 15 years. The mechanism of injury was blunt injury, with 12 motor vehicle accidents (including pedestrians or bicyclists hit by car), 2 falls, and 1 accident at work. After trauma, all subjects were admitted to the intensive care and underwent initial stabilization in the emergency room. When indicated, craniotomies were performed for evacuation of intracranial hematomas. The main inclusion criteria were a Glasgow Coma Scale (GCS) score ≤ 8 following initial resuscitation and the occurrence of an increase in neurological deficit or a deepening of the level of consciousness until death. The main exclusion criteria were history of previous neurologic disease or TBI and of recreational abuse of psychoactive compounds. Abusive head trauma was also excluded. Toxicological analyses for the most common drugs of abuse (cocaine, heroin, amphetamine, methadone and cannabinoids) were negative for all subjects included in the study. As CTRL, we selected subjects died from sudden cardiac death (n = 5). As second control group, we included subjects died from SICH (n = 5).

### Technical details

For all subjects included in the study, autopsy was performed between 24 and 72 h after death. To avoid the progression of transformative phenomena, bodies were kept in cold storage room (−5 °C) until autopsy. Standard sample blocks were taken from the area of focal damage (if macroscopically visible), the cerebral cortex, white and grey matters, basal ganglia, thalami, callosum and the brainstem. In each case, the tissue samples were fixed in 10% formalin for 48 h and then processed and embedded in paraffin.

### Histological and immunohistochemical study

For each case, total sections of about 4 µm thickness were cut and stained with haematoxylin and eosin (H&E). Immunohistochemical investigation was performed as previously described^26, 27^, using antibodies against one or a combination of the following markers: NOX2 (1:50, Santa Cruz, California), 8OHdG (1:10, JaICA, Japan), Neun (1:1000, Abcam, Cambridge, United Kingdom), MAC387 (1:200, Santa Cruz, California), GFAP (1:300, Santa Cruz, California), GAD67 (1:2000, Abcam, United Kingdom), VGLUT1 (1:500, Abcam, United Kingdom), DT1 (1:100 Abcam, United Kingdom), nitrotyrosine (NT, 1:600, Santa Cruz, California), iNOS (1:100,Santa Cruz, California), parvalbumin (1:2000 Abcam, United Kingdom), NOX1 (1: 250 Abcam United Kingdom) and NOX4 (1:100 Abcam United Kingdom). iNOS and NOX2 specificity was tested on positive and technical negative controls (Suppl. Fig. 1). To identify the cellular subtype involved in NOX2 increase, double immunohistochemistry was performed using several peroxidase substrates with different colors: Vector NovaRED (red), Vector VIP (purple), Vector SG (blue/grey) and DAB (brown; Vector, Burlingame, CA, USA). A summary of the markers studied with immunohistochemical reaction is provided in Table 1.

**Table 1:** A summary of markers studied with immunohistochemical reaction.

NOX2	NADPH oxidase 2 or Nox2 is a protein that in humans is encoded by the CYBB gene. The protein is a superoxide generating enzyme which forms reactive oxygen species (ROS).
NOX1	NADPH oxidase 1 or Nox1 is a protein that in humans is encoded by NOX1 gene. The protein is responsible for the catalytic one-electron transfer of oxygen to generate superoxide or hydrogen peroxide.
NOX4	NADPH oxidase 4 or Nox4 is a protein that in humans is encoded by the NOX4 gene. The protein is localized to non-phagocytic cells where it acts as an oxygen sensor and catalyses the reduction of molecular oxygen to various ROS.
8OHdG	8-hydroxy-2′-deoxyguanosine (8OHdG) is an oxidized derivative of deoxyguanosine. 8-Oxo-dG is one of the major products of DNA oxidation. Concentrations of 8OHdG within a cell are a measurement of oxidative stress.
Neun	NeuN antibody (Neuronal Nuclei) specifically recognizes the DNA-binding, neuron-specific protein NeuN, which is present in most CNS and PNS neuronal cell types of all vertebrates tested.
MAC387	Glial marker (Macrophages in human brain tissue)
GFAP (Glial Fibrillary Acidic Protein)	The gene GFAP encodes for an intermediate filament protein (50 kDa) of mature astrocytes, which may be used as a marker for distinguishing astrocytes from other glial cells during development of the central nervous system.
GAD67	Gutamic acid decarboxylase (GAD; E.C. 4.1.1.15) is the enzyme responsible for the conversion of glutamic acid to gamma-aminobutyric acid (GABA), the major inhibitory transmitter in higher brain regions. Two molecular forms of GAD (65 kDa and 67 kDa, 64% aa identity between forms) are highly conserved and both forms are expressed in the CNS.
VGLUT1	The vesicular glutamate transporter 1 VGLUT 1, also referred to as BNPI and SLC17A7, was originally identified as a brain specific phosphate transporter. Like the related VGLUT 2, VGLUT 1 is both necessary and sufficient for uptake and storage of glutamate and thus comprises the sole determinant for a glutamatergic phenotype. Both VGLUTs are different from the plasma membrane transporters in that they are driven by a proton electrochemical gradient across the vesicle membrane. VGLUT 1 and VGLUT 2 show complementary expression patterns. Together, they are currently the best markers for glutamatergic nerve terminals and glutamatergic synapses.
DT1	Markers of dopaminergic neurons
Nitrotyrosine	Nitrotyrosine is a product of tyrosine nitration mediated by reactive nitrogen species such as peroxynitrite anion and nitrogen dioxide. Nitrotyrosine is identified as an indicator or marker of cell damage, inflammation as well as NO (nitric oxide) production. Nitrotyrosine is formed in the presence of the active metabolite NO.
iNOS	Nitric oxide synthase, inducible is an enzyme that in humans is encoded by the NOS2 gene.
Parvalbumin	Parvalbumin is a calcium-binding albumin protein with low molecular weight, present in GABAergic interneurons in the nervous system, especially the reticular thalamus, and expressed predominantly by chandelier and basket cells in the cortex.

Sections were counterstained with methyl green, dehydrated, coverslipped and observed in a Leica DM6000 optical microscope (Leica, Cambridge, UK). Quantification of NOX2, 8OHdG, PV, iNOS and NT positive stained cells was performed using the ImageJ software (imagej.nih.gov/ij/) and expressed as number of positive stained cells/analysed area.

### Statistical analysis

Data were analysed using the GraphPad Prism 5 software for Windows (La Jolla, CA, USA). Data were checked for normality by Bartlett test and analyzed by One-way analysis of variance (ANOVA), followed by Tukey’s post hoc test. For all tests, a P value < 0.05 was considered statistically significant.

### Data availability

The datasets generated during and/or analysed during the current study are available from the corresponding author on reasonable request.

### Compliance with Ethical Standards

This study was performed by using human *post mortem* brain samples, collected during autopsies ordered by the prosecutor and used after the end of the investigations. According to the Italian law, no authorizations from the ethics committee regarding the use of these *post mortem* brain samples were required.

## Electronic supplementary material

Supplementary Figure 1

Supplementary Figure 2
