# Supplementary material for: The NADPH oxidase NOX2 mediates loss of parvalbumin interneurons in traumatic brain injury: human autoptic immunohistochemical evidence
Source: Sci Rep. 2017 Aug 18;7:8752. doi: 10.1038/s41598-017-09202-4 (PMC5562735; doi:10.1038/s41598-017-09202-4)
Supplement: Supplementary file 1 — Supplementary Figure 1 [file 41598_2017_9202_MOESM1_ESM.pdf]

The NADPH oxidase NOX2 mediates loss of parvalbumin interneurons in traumatic brain injury: human autopic immunohistochemical evidence

Stefania Schiavone§, Margherita Neri§, Luigia Trabace#, Emanuela Turillazzi#*

§These authors equally contributed

#These authors equally contributed

*Corresponding Author

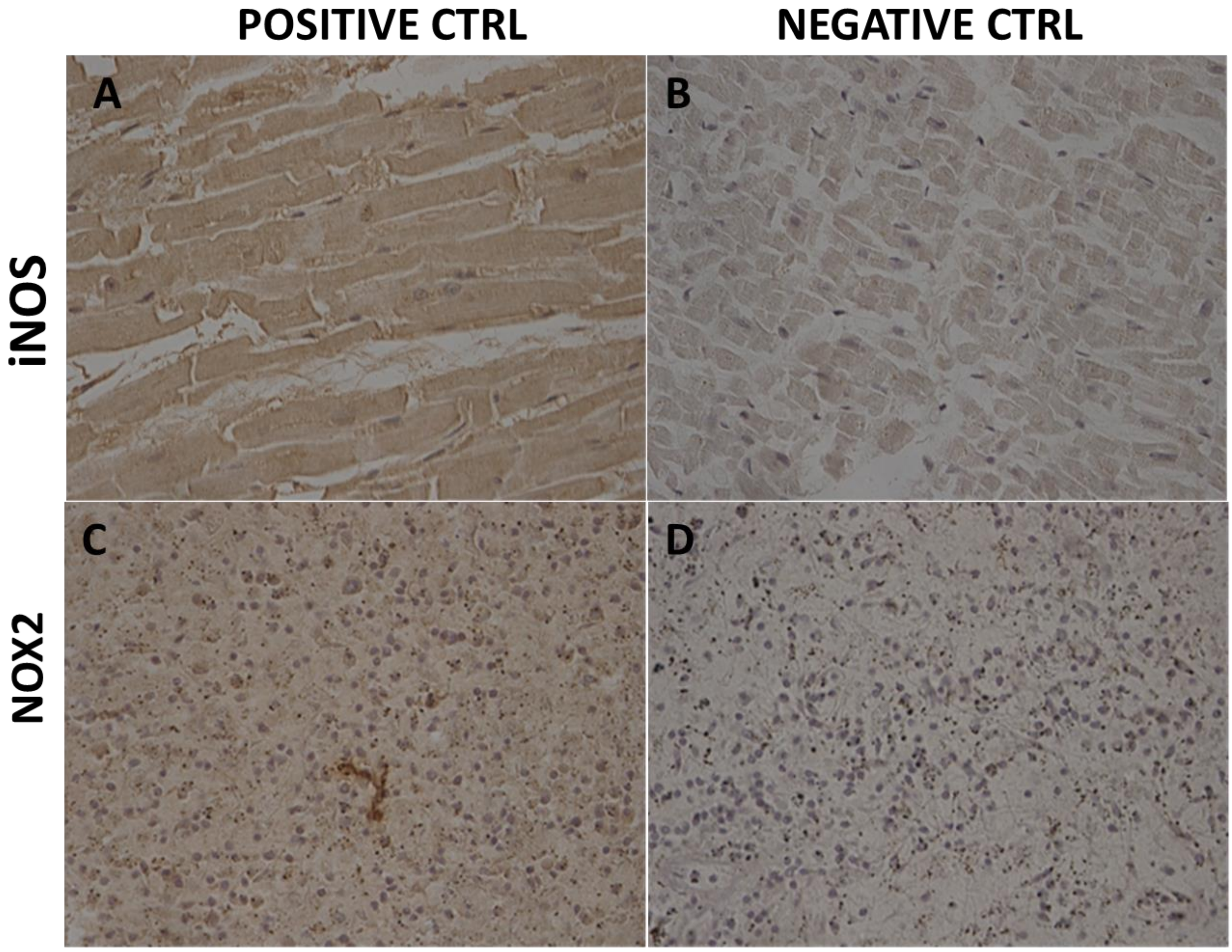

Supplementary Fig. 1: iNOS and NOX2 immunostaining specificity test

A) Representative image of iNOS immunostaining on heart (positive control).

B) Representative image of iNOS immunostaining on heart without primary antibody anti-iNOS (technical negative control).

C) Representative image of NOX2 immunostaining on spleen (positive control).

D) Representative image of NOX2 immunostaining on spleen without primary antibody anti-NOX2 (technical negative control).
