# Supplementary material for: The NADPH oxidase NOX2 mediates loss of parvalbumin interneurons in traumatic brain injury: human autoptic immunohistochemical evidence
Source: Sci Rep. 2017 Aug 18;7:8752. doi: 10.1038/s41598-017-09202-4 (PMC5562735; doi:10.1038/s41598-017-09202-4)
Supplement: Supplementary file 2 — Supplementary Figure 2 [file 41598_2017_9202_MOESM2_ESM.pdf]

The NADPH oxidase NOX2 mediates loss of parvalbumin interneurons in traumatic brain injury: human autopic immunohistochemical evidence

Stefania Schiavone§, Margherita Neri§, Luigia Trabace#, Emanuela Turillazzi#*

§These authors equally contributed

#These authors equally contributed

*Corresponding Author

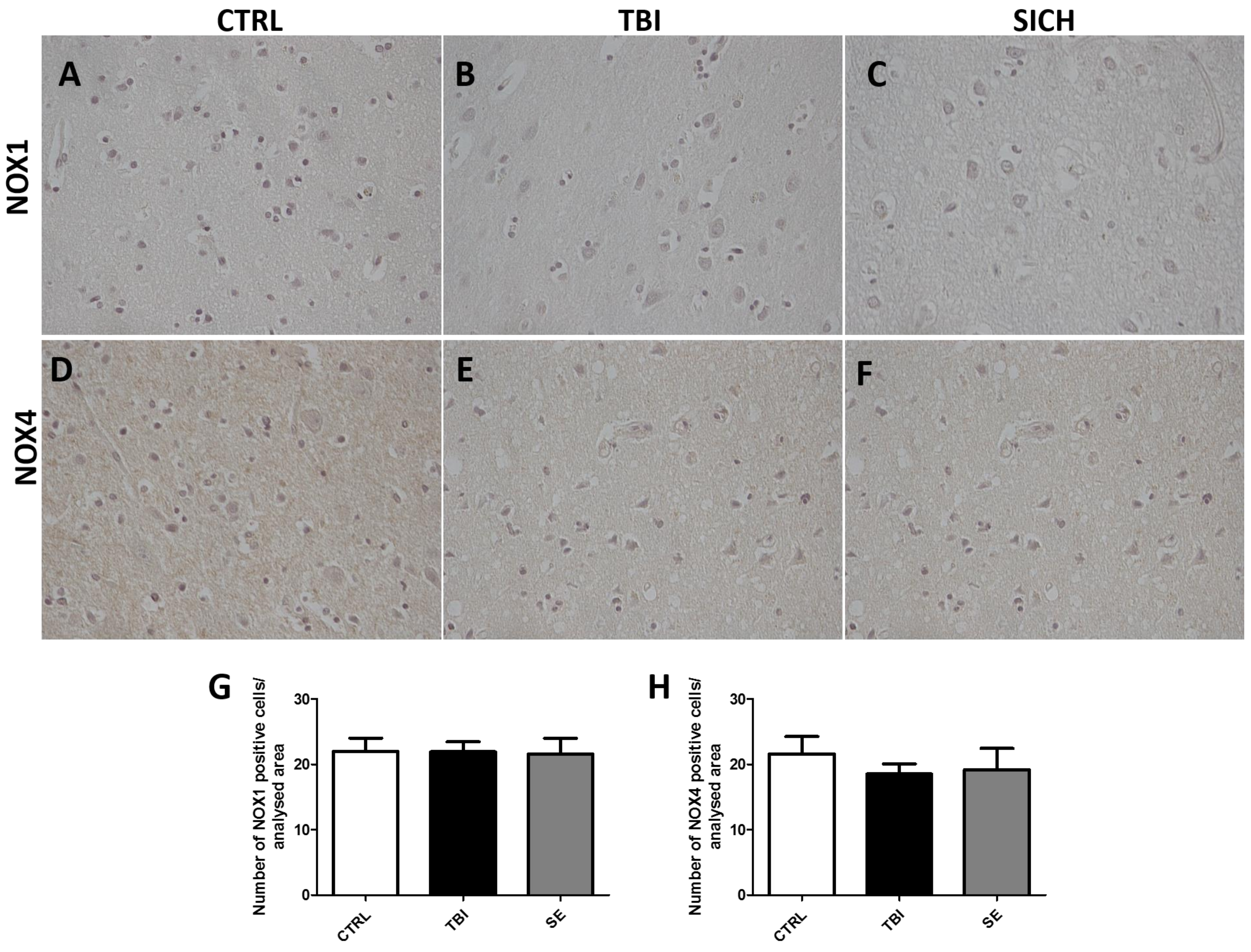

Supplementary Fig. 2: NOX1 and NOX4 expression

(A-C) Representative images of NOX1 immunostaining in the cortex of controls (CTRL, n=5) (A), subjects died following TBI (TBI, n=15) (B) and subjects died following spontaneous intracerebral hemorrhage (SICH, n=5) (C).

(D-F) Representative images of NOX4 immunostaining in the cortex of controls (CTRL, n=5) (D), subjects died following TBI (TBI, n=15) (E) and subjects died following spontaneous intracerebral hemorrhage (SICH, n=5) (F).

(G) Quantification of NOX1-positive-stained cells in the cortex of controls (CTRL, n=5), subjects died following TBI (TBI, n=15) and subjects died spontaneous intracerebral hemorrhage (SICH, n=5). Results are expressed as means ± s.e.m. One-way ANOVA, followed by Tukey's post hoc test, F= 0,008367; P= 0,8332.

(H) Quantification of NOX4-positive-stained cells in the cortex of controls (CTRL, n=5), subjects died following TBI (TBI, n=15) and subjects died spontaneous intracerebral hemorrhage (SICH, n=5). Results are expressed as means ± s.e.m. One-way ANOVA, followed by Tukey's post hoc test, F= 0,4614; P= 0,6364.
